# Development and validation of the mental health professional culture inventory

**DOI:** 10.1017/S2045796019000787

**Published:** 2019-12-16

**Authors:** F. Rapisarda, M. Corbière, A. D. Lesage, L. De Benedictis, J. F. Pelletier, A. Felx, Y. Leblanc, M. Vallarino, M. Miglioretti

**Affiliations:** 1 Department of Psychology, University of Milano-Bicocca, 1 Piazza dell'Ateneo Nuovo, 20126, Milano, Italy; 2 Department of Education – Career Counselling, Université du Québec à Montréal, Montréal, Canada; 3 CIUSSS de l'Est-de-l’Île-de-Montréal, Institut Universitaire de Santé Mentale de Montréal, Montréal, Canada; 4 Department of Psychiatry, Yale University School of Medicine, Yale Program for Recovery & Community Health, New Haven, CT, USA; 5 Department of Brain and Behavioral Sciences, University of Pavia, Pavia, Italy

**Keywords:** Attitudes, psychometrics, psychiatric services, validation study

## Abstract

**Aims:**

No instrument has been developed to explicitly assess the professional culture of mental health workers interacting with severely mentally ill people in publicly or privately run mental health care services. Because of theoretical and methodological concerns, we designed a self-administered questionnaire to assess the professional culture of mental health services workers. The study aims to validate this tool, named the Mental Health Professional Culture Inventory (MHPCI). The MHPCI adopts the notion of ‘professional culture’ as a hybrid construct between the individual and the organisational level that could be directly associated with the professional practices of mental health workers.

**Methods:**

The MHPCI takes into consideration a multidimensional definition of professional culture and a discrete number of psychometrically derived dimensions related to meaningful professional behaviour. The questionnaire was created and developed by a conjoint Italian-Canadian research team with the purpose of obtaining a fully cross-cultural questionnaire and was pretested in a pilot study. Subsequently, a validation survey was conducted in northern Italy and in Canada (Montreal area, Quebec). Data analysis was conducted in different steps designed to maximise the cross-cultural adaptation of the questionnaire through a recursive procedure consisting of performing a principal component analysis (PCA) on the Italian sample (*N* = 221) and then testing the resulting factorial model on the Canadian sample (*N* = 237). Reliability was also assessed with a test-retest design.

**Results:**

Four dimensions emerged in the PCA and were verified in the confirmatory factor analysis: family involvement, users' sexuality, therapeutic framework and management of aggression risk. All the scales displayed good internal consistency and reliability.

**Conclusions:**

This study suggests the MHPCI could be a valid and reliable instrument to measure the professional behaviour of mental health services workers. The content of the four scales is consistent with the literature on psychosocial rehabilitation, suggesting that the instrument could be used to evaluate staff behaviour regarding four crucial dimensions of mental health care.

## Background

In recent decades, mental health care in middle- and high-income countries has been shifting toward community care and a combination of treatment interventions and rehabilitation practices toward recovery (Thornicroft and Tansella, 1999; Piat and Sabetti, 2009; Chen *et al*., 2013). Thus, mental health services workers are required to update their practice by adopting new professional behaviours and visions of mental health care (Chester *et al*., 2016). However, staff expectations, perceptions and attitudes may foster or hinder the implementation of new practices, such as evidence-based interventions (Schoenwald *et al*., 2008), routine outcome assessment procedures (Trauer *et al*., 2009) and goal setting (Clarke *et al*., 2009). Professional sensemaking (Bloor and Dawson, 1994) is particularly relevant in mental health care, where mental health workers deal with clinical conditions that are socially constructed (Rosenhan, 1973; Weiner, 1975; Eisenberg, 1988; Brown, 1995). Colombo and colleagues (Colombo *et al*., 2003) assert that six models of mental disorder can be systematically identified via the narratives of mental health services workers: medical (organic), social, cognitive-behavioural, psychotherapeutic, family and conspiratorial. Moreover, each model may be classified on the basis of 12 key dimensions that define what a mental disorder is, what should be done about it and how those involved should behave toward each other. Thus, every mental health worker explicitly defines what kind of person should be defined as ‘mentally ill’, how they should be ‘treated’ and how much autonomy ‘should be given’ to him/her according to a system of beliefs and attitudes (Slade, 2009, 8–34).

Consistent with scholars' interest in the dissemination of the recovery model, a number of tools have been developed to assess staff's professional behaviours, attitudes and competencies related to the recovery framework, such as the Recovery Self-Assessment (RSA) and the Recovery Attitude Questionnaire (RAQ, Borkin *et al*., 2000). However, since these instruments are based on the recovery model, they do not assess other professional behaviours that could be relevant for service evaluation or training purposes; moreover, they rely mostly on the measurement of attitudes and beliefs that may poorly predict real professional behaviours (Ginsburg *et al*., 2000).

Professional behaviours may be conceptualised within the construct of ‘professional culture’. The creation of a particular professional group leads to the development of a system of values and knowledge that is shared among members of this group and thus allows for the orientation of professional behaviours (Bloor and Dawson, 1994). These elements constitute the ‘professional culture’ of a particular group, influence their decisions and behaviours and thus contribute to the definition of the organisational culture in which those professionals work. Moreover, for healthcare workers who are used to working in multiprofessional teams, as in mental health services, professional culture is also partially shared among team members, since personal endorsement of the shared culture among in-group members fosters identification with that social group (Rapisarda and Miglioretti, 2019) and with the organisational culture (Schein, 2004). A recent meta-synthesis (Rapisarda and Miglioretti, 2019) identified three interrelated dimensions that contribute to professional culture for the staff of MHSWs, i.e. ‘interpersonal distance with users’, ‘power games’ and ‘professional identity over uncertainty’, along with specific interprofessional differences.

Currently, to our knowledge, no instrument has been developed to explicitly assess the professional culture of mental health workers interacting with severely mentally ill people in publicly or privately run mental health services. Because of theoretical and methodological concerns, we designed a self-administered tool to assess the professional culture of mental health services workers. The study aims to validate this tool, named the Mental Health Professional Culture Inventory (MHPCI). The questionnaire was created and developed by a conjoint Italian-Canadian research team, and every research step, from item creation to data analyses, was conducted in parallel in Italy and Canada. Since the construct of professional culture has not been evaluated for professional categories of mental health services workers, this study will focus essentially on construct validity using exploratory and confirmatory factor analyses.

## Methods

### Development phase

The MHPCI was conceived using a multidimensional definition of professional culture and a discrete number of psychometrically derived dimensions related to meaningful professional behaviour (Lesser *et al*., 2010). The MHPCI has been developed by a conjoint team of researchers from Milan (Italy) and from Montreal (Quebec, Canada) with a clinical background. Northern Italy and Quebec have a mental health system with similar historical trajectories (Thornicroft and Tansella, 1999; Lesage, 2006): the two regions have a public system, financed by public welfare, in which mental health care is delivered mostly in community settings, and have sustained a process of deinstitutionalisation during the second half of the 20th century. The entire development phase, from item creation to data analysis, was conducted together by the conjoint team with the purpose of creating an instrument with good ecological and cultural validity (Beaton *et al*., 2000; Johnson, 2006).

A first draft of the questionnaire was created in 2013 and included 71 items generated through a literature review and suggestions from clinical researchers and professionals in Milan and Montreal. Items were created to describe an array of behaviours, feelings and cognitive assessments that a staff member could experience in his/her work with users, with a preference for items focusing on behaviour, instead of focusing on explicit beliefs. For that reason, most of the items were not suitable for describing the professional culture of staff members who do not work in direct contact with users (e.g., administrative staff). The MHPCI items have been created on the basis of the following issues and constructs:

- personal involvement in the relationship with users (Akerjordet and Severinsson, 2004; Burks and Robbins, 2012; Carpenter-Song and Torrey, 2015);
- collaboration with the social network: working with clients' families and making contact with members of the informal social network (Goodwin and Happell, 2007);
- collaboration with the professional network: cooperation among professionals of different services and interorganisational collaboration could be a delicate issue in the MHS practice (Leutz, 1999);
- evidence-based practices and outcome assessment: attitudes and knowledge regarding evidence-based practice (Lavoie-Tremblay *et al*., 2008; Trauer *et al*., 2009) and goal-setting methodology (Clarke *et al*., 2009);
- users' involvement in the process of setting rules and making decisions;
- management of aggressive behaviour: staff's perception of patient aggression (De Benedictis, *et al*., 2011) and cognitive assessments of challenging behaviours (Lambrechts *et al*., 2010);
- users' sexuality (US): MHWs' attitudes and professional behaviours toward actively broaching sexual issues with clients (Cort *et al*., 2001), including homosexuality (Bowers and Bieschke, 2005);
- spirituality and religion: spirituality may be a relevant issue for users (Wilding *et al*., 2006; Curlin *et al*., 2007): MHWs' attitudes and behaviours toward spirituality in MH care;
- professional identity and recognition: the perceived degree of professional recognition by colleagues.

Italian-to-French translation was performed by a bilingual Canadian co-author of this paper (A.L.), and French-to-Italian back-translation was done by an Italian-Canadian co-author (M.V.). Further Canadian-French revisions were also performed by other co-authors (M.C. and A.F.). In 2014, pilot testing was conducted on a small sample of MHWs in Milan (*n* = 38) and Montreal (*n* = 31). Researchers from the Italian-Canadian conjoint team (F.R., M.M. and A.L.) examined the items' quality and removed items with poor psychometrical properties (i.e. more than 10% of missing responses and abstentions, standardised skewness or kurtosis >1.96). The outcome of this preliminary process was a new draft of the questionnaire, composed of 49 items and available in Italian and French Canadian. This new version was used in the following validation phase.

### Validation phase

The MHPCI was tested using a survey design by enrolling staff members working in public MHSs in Italy and Canada. Participants were enrolled considering the following inclusion criteria: (1) staff working in a unit/service that provides mental health care for people with severe mental illness; staff participating in an internship or a voluntary job; (2) staff who have face-to-face contact with users during working hours and (3) staff from a good variety of mental health settings. To do so, participants were recruited from different services and units, including community mental health centres, residential facilities, day care centres and hospital wards, from public, private and non-profit organisations in Italy and only public MHSs in Canada.

In Italy, participants were enrolled in public mental health departments of northern Italy. Moreover, staff from residential and day care units managed by local non-profit organisations were also included in the survey. In Italy, data collection took place from October 2015 to September 2016. The principal investigator (F.R.) presented the project and confidentiality issues to MHWs during routine staff meetings and allowed them 2 weeks to complete the task. All participants were requested to sign a consent form, approved by the Ethics Committee of the University of Milan – Bicocca. Questionnaires were anonymous, and they were printed and presented to participants separately on different documents. Once completed, participants were asked to place the questionnaires and the consent form into different envelopes.

In Canada, all participants were enrolled from the Centre Intégré Universitaire de Santé et de Services Sociaux (CIUSSS) de l'Est-de-l’Île-de-Montréal – mental health and addiction program in the district of Montreal. In Montreal, the research team (A.L., A.F., L.D.B., J-F.P. and Y.L.), with the help of the human resources staff and the mental health program directory, compiled a list of names and positions of all 1200 staff members and 73 physicians from the mental health and addiction programs. The principal investigator from the Italian team (F.R.) assigned numeric codes to each Canadian staff member. Only investigators knew the participants' codes. The Canadian team (A.F.) explained the project and guaranteed confidentiality to the program managers and unit heads before letters were sent to the selected staff members via random sets of 400 questionnaires. Three sets of 400 questionnaires were sent to the staff, the first in October 2016, the second in December 2016 and the third in February 2017, for a total of 1200 questionnaires.

A research coordinator and a group of researchers managed the mailing of the questionnaires from the list by sending an envelope containing an invitation letter, a consent form and the questionnaire to the randomly selected set of participants. The envelope cover only displayed codes and not staff names. Staff members were free to participate by completing the documents (or not, i.e. refusing to participate) and sent envelopes by internal mail to the attention of the main investigator (A. L.). The research coordinator registered the participants' codes written on the received questionnaire and sent a digital scanned copy of each questionnaire to the Italian principal investigator for data entry.

The study received approval from the Ethics Committees of the University of Milan Bicocca and the CIUSSS de l'Est-de-l'Ile-de-Montréal.

### Data analysis

Database preparation involved the overall inspection of data quality, exclusion of incomplete questionnaires, and replacement of missing values. Participants who did not answer five or more MHPCI items were identified as having ‘incomplete questionnaires’ and consequently were excluded from the analysis of validation. Common characteristics and requirements between professional groups in Italy and in Canada were compared to create comparable professional categories for data analysis. The following professional categories are used: registered nurse, counsellor (includes rehabilitation workers with university degree, i.e. professional educator, occupational therapist and rehabilitation technician), psychologist, psychiatrist, social worker and support worker (including mental health workers without a university degree, i.e. paraprofessional nurses).

Data analysis was conducted in different steps designed to maximise the cross-cultural adaptation of the questionnaire through a recursive procedure. The first step of analysis consisted of a principal component analysis (PCA) with Varimax rotation performed on the Italian sample. A factor loading of 0.40 was established as the lower bound for a variable to be included in the respective factor structure (Vallerand, 1989; Corbière, 2014); i.e., items that scored a factor loading lower than 0.40 on a single dimension were eliminated from the model. Cronbach's α was also obtained for each identified factor to avoid low internal consistency. Next, confirmatory factor analysis (CFA) was run on the Canadian sample to confirm the factor solution that emerged in the Italian PCA. To obtain a well-fitted CFA model, the fit indices are as follows: the comparative fit index (CFI) and Tucker-Lewis index (TLI) had to be higher than 0.90, and the root mean square error of approximation (RMSEA) had to be less than or equal to 0.08 (Corbière, 2014).

The factors' internal consistency and reliability were then tested for both the Italian and Canadian samples. Cronbach's α was obtained for each emerging factor. Test-retest reliability was evaluated by computing correlation indices (Pearson's ρ) for scale scores during a 2-week period.

All data analysis was performed using IBM SPSS Statistics v. 23, except for the CFA, which was performed using R software with the Lavaan package retrieved from the project's website (http://lavaan.ugent.be/index.html).

## Results

From a large sample (*n* = 467), 229 questionnaires were completed in Italy, and 238 were completed in Canada. The response rate was approximately 65% in Italy and 20% in Canada (when considering only comparable professionals and full-time present staff). Only nine participants were excluded for missing data (eight in the Italian sample and one in the Canadian sample). All analyses were performed on a sample of 458 participants (i.e., 221 + 237), whose characteristics are described in Table 1.

**Table 1.** Sample characteristics

	Italy *N* = 221	Canada *N* = 237		Total *N* = 458
Sex				
Female	161 (72.9%)	156 (66.1%)	–	317 (69.4%)
Male	60 (27.1%)	80 (33.9%)	–	140 (30.6%)
*Missing*	0	1 (0.4%)	–	1 (0.3%)
Age				
Mean (s.d.)	45.8 (10.1)	44.6 (12.4)	**	45.2 (11.3)
*Missing*	7 (3.2%)	8 (3.4%)	–	15 (3.3%)
Education				
Professional school	31 (14.1%)	18 (7.6%)	*	49 (10.7%)
High school	36 (16.3%)	60 (25.3%)	*	96 (21.0%)
Bachelor of arts	50 (22.6%)	77 (32.5%)	*	127 (27.7%)
Master's degree	44 (19.9%)	38 (16.0%)	–	82 (17.9%)
Medical specialisation/PhD/Other	56 (25.3%)	41 (27.3%)	–	97 (21.2%)
*Missing*	4 (1.8%)	3 (1.3%)	–	7 (1.5%)
Job				
Nurse	68 (30.8%)	49 (20.7%)	*	117 (25.5%)
Counsellor	69 (31.2%)	54 (22.8%)	*	123 (26.9%)
Psychiatrist	31 (16.7%)	31 (13.5%)	–	69 (15.1%)
Psychologist	17 (7.7%)	16 (6.8%)	–	33 (7.2%)
Social worker	10 (4.5%)	32 (13.5%)	**	42 (9.2%)
Support worker	16 (7.2%)	26 (11.0%)	–	42 (9.2%)
Other	1 (0.5%)	25 (11.8%)	**	26 (7.0%)
*Missing*	3 (1.5%)	3 (1.3%)	–	6 (5.7%)
Years working in the mental health field				
Mean (s.d.)	15.8 (9.3)	17.0 (12.2)	–	16.4 (10.9)
*Missing*	2 (0.9%)	2 (0.8%)	–	(0.9%)
Average weekly working hours				
Mean (s.d.)	33.5 (8.2)	34.8 (8.0)	*	34.1 (8.1)
*Missing*	1 (0.5%)	3 (1.3%)	–	
Work setting				
Home care	15 (6.8%)	12 (5.1%)	–	27 (5.9%)
Mental health centre or day care centre	123 (55.7%)	90 (38.0%)	**	213 (46.5%)
Hospital unit	28 (12.7%)	77 (32.5)	**	105 (22.9%)
Residential facility	54 (24.4%)	45 (19.0%)	–	99 (21.6%)
Other	1 (0.5%)	6 (2.5%)	–	7 (1.6%)
*Missing*	0	7 (3.0)	**	7 (1.6%)

Statistically significant differences (*χ*^2^ or *T* test): *p < 0.05; **p < 0.01.

Participants were mostly female (69.4%), with an average age of 45.2 years (s.d. = 11.3), 66.8% of them had a university degree, and they had worked in the mental health field for an average of 16.4 years (s.d. = 10.4). Among the professional roles, nurses, psychiatrists (including five residents in training) and counsellors were the most represented categories, constituting 64.6% of the sample. Significant differences (*p* < 0.05) between the Italian and Canadian samples were found in age, education levels, professional roles and weekly working hours (Table 1).

The results of the PCA conducted on the Italian sample are displayed in Table 2.

**Table 2.** PCA of the BMHPCI (Italian sample)

	1	2	3	4	Explained variance
Component 1: ‘Family involvement’					15.4%
I keep regular contacts with families	0.88				
I foster family members' involvement	0.81				
I can rely on families	0.66				
I can really improve user's quality of life when I intervene on family and social context	0.53				
Component 2: ‘User's sexuality’					15.3%
I ask users to talk about their sexuality		0.79			
I talk with users about sexual education		0.74			
I talk with colleagues about users' sexuality		0.73			
I help users to accept being homosexual		0.63			
Component 3: ‘Therapeutic Framework’					13.2%
I can integrate protocols and manuals with my way of working			0.75		
I consider other services staff's views about users even when they are different from mine			0.71		
I find the opinion from previous clinicians useful for setting up treatment plans			0.62		
My work with users is based on well documented models			0.58		
Component 4: ‘Management of Aggression Risk’					12.5%
I'm on alert to cope with aggression				0.77	
I avoid being alone with users with severe mental illness				0.74	
I am subjected to threats and aggression				0.70	

Factor loadings <0.30 were not displayed in table.

Thirty-four items presented saturation coefficients <0.40 and were removed from the model; the resulting solution, which explained 56.4% of the variance, included 15 items and were grouped in the following four components:

1. *Family involvement* (FI), four items that describe MHWs' attitudes toward the involvement of users' families and informal networks in the care process;
2. US, four items that describe how frequently MHWs deal with US issues in their practice;
3. *Therapeutic framework* (TF), four items describing the adoption of theoretical models and protocols in the professional's practice and collaboration with staff from other services;
4. *Management of aggression risk* (MAR), three items about the risk of being assaulted by clients.

While some of the removed items dealt with topics of the above components, most of them referred to different topics (such as personal involvement in the relationship with users, users' involvement in the process of rule setting and decision-making, spirituality and religion) that did not emerge with a clear pattern of loadings.

**Fig. 1.**
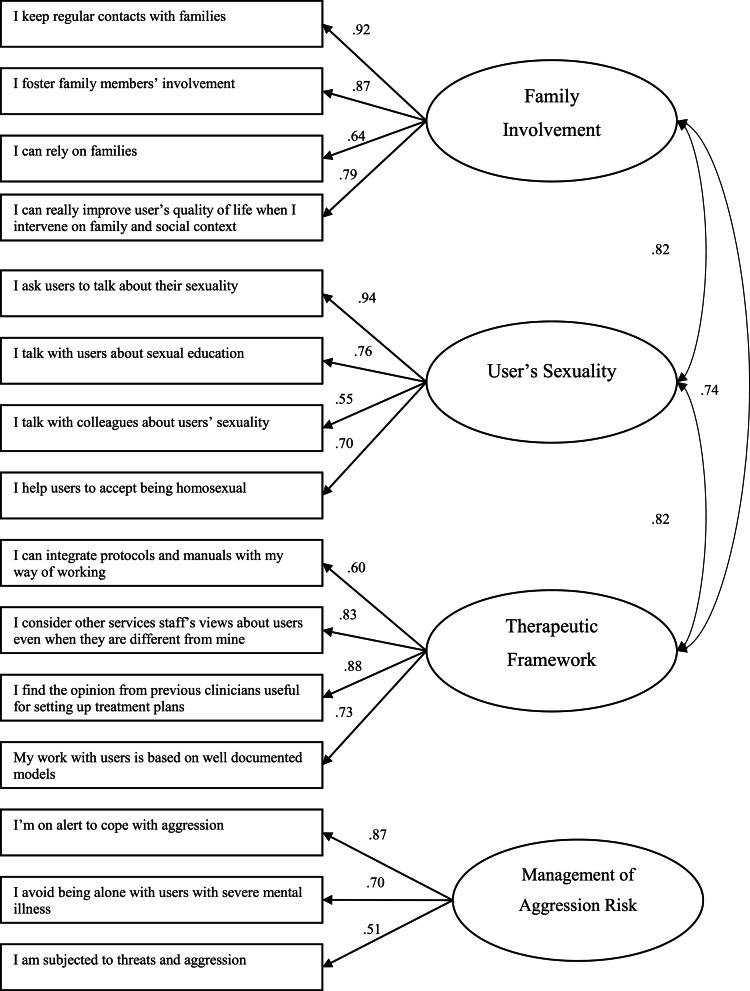
Confirmatory factor analysis (Canadian sample).

Components that emerged in the PCA were also confirmed in the CFA ran on the Canadian sample (Figure 1): the model had good fit indices (CFI = 0.90; TLI = 0.90; RMSEA = 0.072), and a significant correlation among FI, US and TS was observed.

The scale validity and reliability scores are presented in Table 3. Cronbach's α indicated an acceptable level of internal consistency for all scales in both samples, with a borderline value of 0.59 for the *MAR* scale in the Canadian sample. The correlation coefficients for test-retest reliability ranged from 0.69 to 0.91. As the satisfaction threshold is generally set at 0.60 (Vallerand, 1989), the four dimensions of the MHPCI can be regarded as stable measures at a 2-week time interval. Moreover, a statistically significant correlation among FI, US and TF was found in both samples, with only a slightly significant difference in the correlation between FI and MAR in the Italian sample.

**Table 3.** Scales reliability and internal consistency

	FI	US	TF	MAR
1. FI	*α* = 0.82; 0.85 *r* = 0.88; 0.84	0.62**	0.58**	0.05
2. US	0.36**	*α* = 0.62; 0.72 *r* = 0.71; 0.81	0.46**	0.03
3. TF	0.30**	0.29**	*α* = 0.60; 0.63 *r* = 0.89; 0.64	0.10
4. MAR	0.18**	0.03	−0.03	*α* = 0.63; 0.59 *r* = 0.84; 0.88

*α* = Chronbach's α values in the Italian (first) and Canadian (latter) sample.

*r* = test-restest correlation coefficients in the Italian (first) and Canadian (latter) sample.

Below the diagonal, correlation coefficients (Pearson's *r*) between subscales for the Italian sample are presented, and above the diagonal, correlation coefficients between subscales for the Canadian sample.

**p < 0.01.

## Discussion

The study's aim was to develop a new questionnaire, the MHPCI, to assess professional behaviours related to the professional cultures of mental health services workers. The results indicate that the MHPCI has good internal consistency, cross-cultural validity and short time stability. Although the ten clusters were conceived *a priori,* both the explorative PCA and the CFA obtained four dimensions, including 15 items: three scales (*FI*; *US* and *MAR*) confirmed the clusters hypothesised by the researchers during the development process; one scale entitled *TF* emerged in the PCA and was confirmed by the CFA. To our knowledge, no existing questionnaire covers all four dimensions and adopts a behavioural approach to assess professional culture.

The dimensions that constitute the final version of the MHPCI include constructs that are well known in research and clinical practice. FI has been progressively better recognised in the psychosocial rehabilitation literature (Hall, 1999; Goodwin and Happell, 2007). In one survey of psychiatric foster families and residents of group homes, the latter nominated their own family as the one who believed most in their recovery, and the Competency Assessment Instrument (Chinman *et al*., 2003) considers FI as a core competency of mental health services workers. The *US* scale is an original scale, since current tools that assess professional behaviour do not include items related to sexuality. However, items about US were included in the Camberwell Assessment of Needs (Phelan *et al*., 1995) but not in previous formal needs assessment questionnaires (Comtois *et al*., 1998). In the latter study, sexuality emerged as the fifth most important problem reported by patients, and it was not part of the needs assessment questionnaire. The *MAR* scale refers to an issue that is not included in the most frequently used recovery questionnaires (such as the RSA or the RAQ), reflecting adherence to set values toward the patient, instead of the realities of daily practice, such as those experienced by ward nurses or relative caregivers of patients (Lemelin *et al*., 2009; Edward *et al*., 2016). Even though the internal consistency of this scale was slightly below a satisfactory level, this value could be due to the small number of items (only three items), explaining the low internal consistency (Corbière and Fraccaroli, 2014). Previously a silenced dimension, it may become a point of negotiation between patients, staff and relatives, who are all potential ‘victims’ of violence by one other (Guglielmetti *et al*., 2016; Onwumere *et al*., 2018).

Finally, the *TF* scale groups together items from two different semantic clusters, i.e., collaboration with formal network and evidence-based practices. Even though this scale combines topics that are already known in the literature and that have been studied with specific scales (Clarke *et al*., 2009; Trauer *et al*., 2009; Lavoie-Tremblay *et al*., 2008), our findings suggest the presence of a common construct that may refer to professionalism.

### Strengths and limitations

The four scales of the MHPCI were validated through a bottom-up approach: 49 items were tested in two samples from different countries, and four factors emerged in both samples. Moreover, participants worked in a wide range of mental health settings, from hospital wards to community teams, with different professional roles and backgrounds. This variety of professionals may ensure that the emerging dimensions have cross-professional and cross-cultural validity that allow the tool to be used across all types of MHSs and diverse professionals, which is consistent with the study's aim.

This study does have some methodological limitations. First, the item generation procedure was not adequately sustained by conceptual mapping. Moreover, since professional culture has not been studied in mental health care, researchers could only hypothesise the constructs by relying on a free interpretation of the existing literature. The lack of conceptual mapping in item generation could also explain the elimination of a large percentage of items during the PCA. Since the elimination involved some specific topics and not others, it could be asserted that the remaining factors are better suited to measure professional culture than other topics that, although they are already present in the literature, do not fit well in a multifactorial solution. Second, response rates were different in the two countries, reflecting differences in administrative procedures: in Italy, researchers presented the questionnaire to the research team during regular staff meetings, whereas in Canada, questionnaires were sent via regular mail. Despite differences in the response rate, the two samples were comparable in terms of participants' characteristics. Third, convergent and discriminant validity were not evaluated (Corbière and Fraccaroli, 2014) since no other instruments were available in Italian and French to perform correlational analysis.

### Conclusion and future research

Despite the limitations mentioned above, this study suggests that the MHPCI could be considered a valid and reliable instrument to determine the professional behaviours of mental health services workers. The content of the four scales is consistent with the existing literature on psychosocial rehabilitation, suggesting that the instrument could be used to evaluate staff behaviour regarding four crucial dimensions of mental health care: (1) working with the microsocial environment (families); (2) valuing the principles of psychosocial rehabilitation and evidence-based practices; (3) addressing the intimate life needs of patients and (4) promoting a culture of safety in the work environment in which patients, staff and relatives interact with each other.

Further research is needed to test the differences between specific professional groups (i.e., psychiatrists *v.* nurses) and to investigate the determinants of professional culture, such as age, experience in mental health settings and training. A further step would be the implementation of the tool to evaluate training programs and user satisfaction.

## Availability of data and materials

The data on which this manuscript is based are available upon request. The first author can be contacted with a request within 5 years after the article publication. Researchers are requested to submit a research plan, describing its background, scope and an outline the analyses. Upon approval, a written agreement will be signed stating that the data will only be used for addressing the agreed-upon research scope and not for other purposes.
